# The Effect of Surgeon Volume on the Outcome of Laser Vaporization: A Single-Center Retrospective Study

**DOI:** 10.3390/curroncol29050302

**Published:** 2022-05-23

**Authors:** Michihide Maeda, Tsuyoshi Hisa, Shinya Matsuzaki, Misooja Lee, Seiji Mabuchi, Shoji Kamiura

**Affiliations:** 1 Department of Gynecology, Osaka International Cancer Institute, Osaka 541-8567, Japan; michihide.maeda@oici.jp (M.M.); hisa-tu@mc.pref.osaka.jp (T.H.); shinya.matsuzaki@oici.jp (S.M.); seiji.mabuchi@oici.jp (S.M.); 2 Department of Forensic Medicine, School of Medicine, Kindai University, Osaka 577-8502, Japan; misooja-88@umin.ac.jp

**Keywords:** carbon dioxide laser vaporization, cervical intraepithelial neoplasia, prognostic factors, recurrence, surgeon volume

## Abstract

Although laser vaporization is a popular minimally invasive treatment for cervical intraepithelial neoplasia (CIN), factors influencing CIN recurrence are understudied. Moreover, the effect of surgeon volume on patients’ prognosis after laser vaporization for CIN is unknown. This single-center retrospective study evaluated the predictive value of surgeon volume and patient characteristics for laser vaporization outcomes in women with pathologically confirmed CIN2. Histologically confirmed CIN2 or higher grade after laser vaporization was defined as persistent or recurrent. Various patient characteristics were compared between women with and those without recurrence to examine the predictive factors for laser vaporization. There were 270 patients with a median age of 36 (18–60) years. The median follow-up period was 25 (6–75.5) months and the median period between treatment and persistence or recurrence was 17 (1.5–69) months. The median annual number of procedures for all seven surgeons was 7.8. There were 38 patients (14.1%) with persistent or recurrent lesions—24 had CIN2, 13 had CIN3, and one had adenocarcinoma in situ. Patient age, body mass index, surgeon volume, and history of prior CIN treatment or invasive cervical cancer were not significantly correlated with lesion persistence or recurrence. In conclusion, laser vaporization has comparable success rates and is a feasible treatment for both low- and high-volume surgeons.

## 1. Introduction

Cervical intraepithelial neoplasia (CIN) is a premalignant lesion of the uterine cervix histologically graded as CIN1, CIN2, or CIN3 [1,2]. The aim of CIN management is to prevent possible progression to invasive cancer while avoiding overtreatment, because lesions can spontaneously regress, and treatment can have adverse effects on future fertility and obstetric outcomes [3]. Women with a histological diagnosis of CIN2 are recommended to undergo treatment or active surveillance [4,5]. Treatment options include excisional (cold-knife, needle, or laser conization, large loop excision) and ablative procedures (cryocoagulation, laser vaporization) [6].

With excisional treatment, a sample is available for histological examination, enabling the assessment of the margins to confirm whether the excision is complete [7]; however, it is associated with increased risk of preterm delivery [3,8]. Thus, less invasive treatment for CIN2 is preferred, such as laser vaporization, which is not associated with adverse outcomes in future pregnancies [9,10]. Nevertheless, laser vaporization also has certain drawbacks: a lack of histological examination and a high rate of treatment failure (approximately 10–20%) [11,12]. Women treated for CIN with laser vaporization were found to have a higher risk of recurrence or progression to invasive cancer for years after treatment [13].

Based on this background, a predictive factor for the success of treatment after laser vaporization for CIN2 is warranted. Recent studies have reported that surgeon and hospital case volumes affect perioperative morbidity and mortality, surgical outcomes, and cost-effectiveness [14,15,16]. As laser vaporization is not widely performed owing to the cost of its equipment; we believe that the examination of the effect of surgeon volume on the treatment outcome of laser vaporization may be useful. We believe that the clinical outcomes of patients undergoing laser vaporization may be favorable if they are operated by high-volume surgeons in comparison to low-volume surgeons. We hypothesized that if surgeon volume is associated with the success rate of laser vaporization, some surgical techniques such as ablation depth may affect the success rate [11]. On the contrary, if no significant relationship exists, laser vaporization for CIN may be feasible to be performed by most surgeons.

Therefore, this study aimed to examine the predictive factors for the success of laser vaporization for CIN2, including the effect of annual surgeon volume.

## 2. Materials and Methods

### 2.1. Study Design and Patient Eligibility

This was a single-center retrospective observational cohort study that included women with pathologically confirmed CIN2, who were treated with carbon dioxide laser vaporization at the Osaka International Cancer Institute between January 2013 and March 2019. Patients that met one of the following criteria were identified: (1) patients with CIN2 having high-risk human papillomavirus (HPV) infection; (2) persistence of CIN2; or (3) laser vaporization at the patient preference. All procedures were performed by an experienced gynecologist in an outpatient setting using AcuPulse (Lumenis Ltd., Yokneam, Israel) with a power of 20 W in continuous mode.

Inclusion criteria of this study were as follows: (1) women with CIN2 treated with laser vaporization between the ages of 18 and 60 years and (2) those whose follow-up period was at least 6 or more months. Exclusion criteria were as follows: women with (1) CIN3 or higher grade of the disease; (2) age > 60 years; (3) first follow-up more than 12 months after laser vaporization; (4) follow-up period of less than 6 months; (5) suspected invasive cancer; (6) incomplete patient data; (7) positive pregnancy test; and (8) use of immunosuppressants. 

This study was approved by the Institutional Review Board of the Osaka International Cancer Institute (approval number: 21129) and is reported in line with the STROBE guidelines [17].

### 2.2. Clinical Variables

Patient demographics, tumor characteristics, treatment type, and treatment outcomes were extracted from the database. Patient demographics included age (<50 and ≥50 years), year of diagnosis, parity, body mass index (BMI) (≤25 and >25), cigarette smoking status, HPV infection status, menopause, use of hormonal contraceptives, surgeon volume (<10 and ≥10 laser vaporization procedures per year), last follow-up, complications, and prior treatment history of CIN.

### 2.3. Outcome Measures

Primary outcome measures included persistence or recurrence of CIN2 lesions, defined as histologically confirmed CIN2 or more severe disease after laser vaporization. Secondary outcome measures included the rates of complications and invasive cancer detection after laser vaporization.

### 2.4. Treatment Follow-Up

In our practice, the first follow-up evaluation is performed by liquid-based cervical cytology test within 6 months after laser vaporization. Resampling for cervical cytology is performed 3–6 months after the first cytology test to assess treatment response. 

If the first and second cytology test results were negative for intraepithelial lesions or malignancy, annual cytology testing was performed. If the first or second cytology test result was low-grade squamous intraepithelial lesion or a higher-grade lesion, colposcopic biopsy was performed. In case of histologically confirmed CIN2, the patient was recommended repeat treatment. If CIN1 or less was confirmed, surveillance was continued with cervical cytology testing every 3–6 months until the CIN lesion regressed.

### 2.5. Study Definitions

Annual surgeon volume for laser vaporization was defined as the average number of procedures a surgeon performed per year. In this study, the surgeon volume was divided into high (three surgeons) and low (four surgeons) as previously described [18]. 

Based on the presence of CIN2 persistence or recurrence, patients were divided in two groups: recurrence and nonrecurrence. We evaluated the interval between diagnosis and persistence or recurrence from any cause (all-cause). Women that did not have recurrence at the last follow-up were excluded. For women who underwent repeated laser vaporization treatments, the first treatment within the study period was included.

### 2.6. Statistical Analysis

The primary objective of the analysis was to examine the predictive factors for the success of laser vaporization for CIN2. In the analysis between women with recurrence versus women without recurrence, the chi-square test or Fisher’s exact test was used to analyze the differences in patient characteristics between the groups. The Mann–Whitney U test was used to compare the nonparametric data between the two groups. Additionally, women were divided into two groups to examine the effect of surgeon volume on the treatment outcome (women treated by high-volume surgeons versus women treated by low-volume surgeons).

The Kaplan–Meier method was used to construct survival curves, and differences between the groups were assessed using the log-rank test. The effect size of statistical significance was expressed as odds ratio (OR) and 95% confidence interval (CI). Risk factors were analyzed using the Cox proportional hazards model with a hazard ratio (HR) and 95% CI. All tests were two-tailed and a *p*-value of less than 0.05 was considered statistically significant. EZR (ver. 1.41, Jichi Medical University Saitama Medical Center, Saitama, Japan) was used for the analyses [19].

## 3. Results

A total of 270 patients with pathologically confirmed CIN2 were included in this study. The median age and BMI were 36 years and 20.5 kg/m^2^, respectively. Most women were nulligravid (*n* = 187, 69.3%) and more than half had a positive high-risk HPV test (*n* = 168, 62.2%). The baseline characteristics are shown in Table 1. 

During the study period, seven surgeons performed laser vaporization. The mean annual number of procedures for each was 18.4, 16.6, 10.3, 7.8, 5.9, 5.0 and 1; while the median annual number of procedures for all seven surgeons was 7.8. Hence, there were three surgeons who belonged to the high-volume category and four surgeons to the low-volume category.

The median follow-up period was 25 (6–75.5) months and the loss to follow-up rate was 10.7% within the first year. The median time between treatment and first follow-up evaluation was 57 (23–230) days, while the median time between treatment and persistence or recurrence was 17 (1.5–69) months.

Among the 270 patients, 38 (14.1%) experienced persistence or recurrence with a histological diagnosis of CIN2 (24/38), CIN3 (13/38), or adenocarcinoma in situ (1/38). For salvage treatment, laser vaporization was performed in twenty patients, conization in fifteen, hysterectomy in one, and active surveillance in two patients. 

Regarding complications, postoperative bleeding was observed in twelve patients (4.4%) and infection in one patient (0.4%). There were no cases of invasive cancer during the follow-up period.

There were no significant differences between the recurrence and nonrecurrence groups regarding age, BMI, parity, cigarette smoking status, menopause, use of hormonal contraception, history of prior CIN treatment, and surgeon volume (Table 2). 

**Figure 1:**
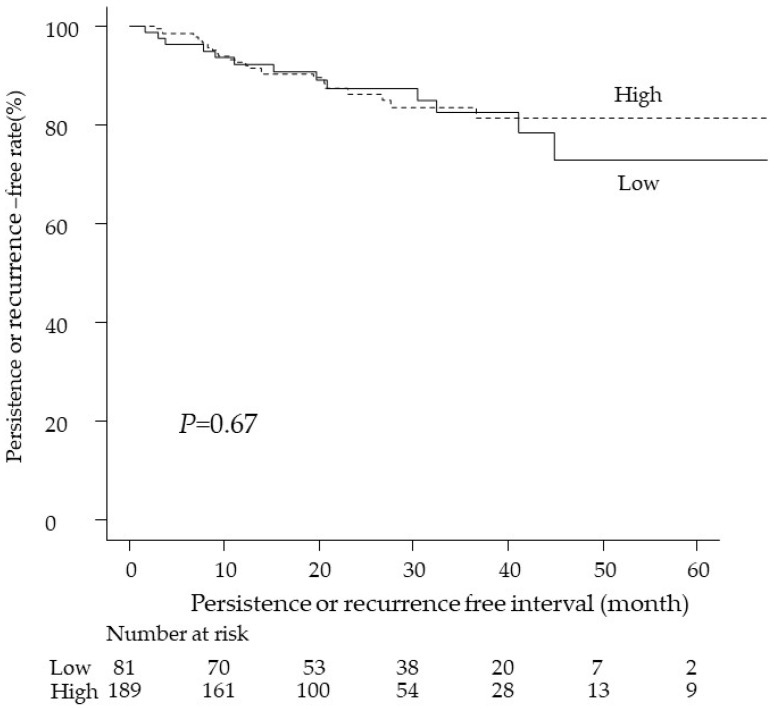
Kaplan–Meier time-to-persistence or recurrence curves based on surgeon volume. The dotted line represents the persistence or recurrence curve of women treated by low-volume surgeons, while the solid line represents the curve of women treated by high-volume surgeons. The Cox proportional hazard regression model was used to determine the *p*-value.

In the comparison of patients’ characteristics and treatment outcome based on surgeon volume, there were no significant differences in any of the evaluated variables (Table 3). Furthermore, the Kaplan–Meier analysis showed no significant difference in the persistence- or recurrence-free interval between high- and low-volume surgeons (*p* = 0.67; Figure 1).

In the univariate and multivariate analyses of possible predictive factors, none of the included variables (age, BMI, surgeon volume, and history of prior CIN treatment or invasive cervical cancer) were significantly correlated with CIN2 persistence or recurrence after laser vaporization (Table 4).

## 4. Discussion

The principal findings of this study were as follows: (1) the surgeon volume was not related with the patients’ outcome after laser vaporization for CIN, and (2) the morbidity of laser vaporization appeared to be comparable for both low- and high-volume surgeons.

Several studies have reported the efficacy and feasibility of laser vaporization for CIN [11,20,21]. As with this procedure there is no surgical specimen to evaluate, most studies have investigated the success rates, complications, and risk factors for recurrence to compensate for this drawback. However, in our humble opinion, we believe that determining prognostic factors for recurrence after laser vaporization may be more useful.

A Japanese nationwide study examined the results of the histological examination after conization in women with CIN1 and CIN2 (*n* = 1536), and found that nearly half of the women (*n* = 719, 45.0%) were diagnosed with CIN3 and approximately 3% (*n* = 41, 2.7%) were diagnosed with invasive cervical cancer after conization [22]. These results suggest that careful evaluation before laser vaporization is essential. 

In previous studies, there have been a few cases of recurrence of invasive cervical cancer after laser vaporization in women with CIN2. In a single-center retrospective study, 1 out of 485 patients (0.2%) experienced recurrence of invasive cervical cancer after laser vaporization for CIN2, and another study showed that none of the 141 patients experienced recurrence of invasive cancer [11,20]. However, this study did not examine the surgeon volume of laser vaporization. In the current study, there were no cases of invasive cancer during the follow-up period regardless of the surgeon volume. This result suggests that laser vaporization is a feasible treatment for both low-and high-volume surgeons.

The essential concept of the volume–outcome relationship was initially reported in 1979 to regionalize patient care after complex surgeries and to improve surgical outcomes [23]. Studies over the past decade have clearly shown a relationship between high-volume surgeons and hospital surgical volume and improved surgical outcomes in women undergoing gynecologic surgical procedures [24]. For laparoscopic hysterectomy for benign indications, patients operated on by high-volume surgeons and those treated at high-volume centers were less likely to experience complications [15]. A systematic review that examined the impact of gynecological surgeon volumes on patient outcomes showed that gynecologists performing procedures approximately once a month or less had higher rates of adverse surgical outcomes [25]. 

While the relationship between surgeon volume and gynecologic procedures is robust, it remains understudied for laser vaporization. We performed a systematic literature search on PubMed and Scopus from their inception to 31 January 2021, using keywords related to laser vaporization and CIN. This systematic search indicated that the effect of surgeon volume on the outcome of laser vaporization has not been reported. 

The present study did not identify a significant relationship between surgeon volume and the success rate of laser vaporization. We believe that this result is because laser vaporization is a relatively simple procedure. Namely, in complex surgery such as radical hysterectomy, high-volume surgeons were related to lower perioperative morbidity, transfusion rates, and shorter hospital stay [26]. However, no volume–outcome relationship was observed for laparoscopic hysterectomy for early endometrial cancer [27]. 

Given the limited number of laser vaporizations performed due to the high cost of the equipment, a negative relationship between surgeon’s volume and the treatment outcome may be helpful to perform laser vaporization for CIN in a general hospital [28]. On the contrary, the minimum volume requirement of laser vaporization for CIN is still unclear; thus, further studies are warranted to identify the minimum volume standards for performing laser vaporization in cases of CIN [29,30].

The strength of this study is that it is likely the first to examine the effect of surgeon volume on the outcome of laser vaporization for CIN. The feasibility of laser vaporization was confirmed for both low- and high-volume surgeons. 

The salient limitations of this study are as follows. First, it was a single-center retrospective study; thus, unmeasured bias may exist. For instance, possible confounders missing in the analysis included comorbidity, HPV status, subsequent treatment for recurrence, and that the decision-making process for laser vaporization was unknown in some patients. Furthermore, the study included a limited number of patients and was considered to be underpowered. Second, the follow-up period was relatively short. In some cases, follow-up was performed at another clinic when both the first and second cytology were negative for intraepithelial lesions or malignancy after laser vaporization. In general, patients were referred to our institution when recurrence was suspected. Therefore, we believe that this point is not a critical bias in this study. 

Third, reproductive outcomes were not determined in this study, but may be a key outcome measure for laser vaporization in young women. Fourth, the surgeon volume in this study had a relatively narrow range. Moreover, all surgeons were associated with a high-volume center; thus, annual treatment volumes may be higher than those in general hospitals. In fact, the rate of treatment failure in this study (14.1%) is consistent with those of previous studies (approximately 10–20%), reported from high-volume centers [11,12]. Nevertheless, this point may cause a severe bias of this study and needs to be recognized. Therefore, future multicenter prospective studies including a larger number of women, many surgeons, and both low- and high-volume centers are warranted to examine the effect of surgeon volume on the outcome of laser vaporization for CIN.

Fourth, there is no clear consensus regarding the definition of a high-volume surgeon. In the previous studies which examined the association between a surgeon’s volume and surgical outcomes, the definition of a high-volume surgeon varied among each study [25]. Since the included cases in this study are limited, we divided surgeons into high- and low-volume, using the median number of annual treatment cases performed by them. As all surgeons in the present study were in the high-volume category, this severely restricted the analyses that could be performed.

Fifth, the difficulty in the diagnosis of CIN2 may have introduced a bias in this study. Notably, the diagnosis of CIN2 in some cases of this study was not confirmed by two independent pathologists. Moreover, a formal independent review of all previous pathology results was not performed and centrally recorded pathological findings were not available in this study. Since most of the features of CIN2 and CIN3 are overlapping, the differentiation between these two is difficult; consequently, in the present study, there may have been cases that could possibly have had a different grade of CIN than that originally diagnosed.

Lastly, the cervical biopsy was not representative of all the affected tissue, and diagnosis in women treated with laser vaporization was not determined by a particular specimen. Therefore, the actual diagnosis of CIN2 is unclear in this study, resulting in a selection bias. Notably, if a malignant disease was misdiagnosed for its histological type or its lymphovascular space invasion, the prognosis of patients may be worse than that actually predicted [31,32,33,34]. Both low- and high-volume surgeons should bear this in mind while performing laser vaporization treatments for CIN.

## 5. Conclusions

Laser vaporization has comparable success rates and is a feasible treatment for both low- and high-volume surgeons.

## Figures and Tables

**Table 1:** Baseline characteristics (*n* = 270).

Patient Characteristics at Baseline	Values
Age, years	36 (18–60)
BMI, kg/m^2^	20.5 (14.8–31.1)
Prior treatment	25 (9.3)
Preoperative cytology	
ASC-US	19 (7.0%)
LSIL	51 (18.9%)
ASC-H	12 (4.4%)
HSIL	187 (69.3%)
AGC-NOS	1 (0.5%)
Parity, *n* (%)	
0	187 (69.3%)
≥1	83 (30.7%)
HPV infection	
Positive	158 (58.5%)
Negative	6 (2.2%)
Not examined	106 (39.3%)
Smoking, *n* (%)	
Yes	120 (44.4%)
No	150 (55.6%)
Use of contraception	
Yes	14 (5.2%)
No	256 (94.8%)
Surgeon volume	
High	189 (70.0%)
Low	81 (30.0%)

Data are presented as median (range) or number (percentage per column). Abbreviations: SD, standard deviation; prior treatment, Prior treatment for CIN; ASC-US, atypical squamous cells of undetermined significance; LSIL, low-grade squamous intraepithelial lesion; HSIL, high-grade squamous intraepithelial lesion; ASC-H, atypical squamous cells, cannot exclude HSIL; AGC-NOS, atypical glandular cells not otherwise specified.

**Table 2:** Comparison of baseline characteristics between the recurrence and nonrecurrence groups.

Persist or Rec	Yes	No	*p*-Value
	*n* = 38	*n* = 232	
Age			
<50	34 (14.3%)	208 (85.7%)	0.99
≥50	4 (14.2%)	24 (85.8%)	
Parity			
0	23 (12.3%)	164 (87.3%)	0.26
≥1	15 (18.1%)	68 (81.9%)	
BMI			
≤25	37 (14.6%)	217 (85.4%)	0.71
>25	1 (6.3%)	15 (93.7%)	
Smoking			
Yes	16 (13.3%)	104 (86.7%)	0.86
No	22 (44%)	128 (56%)	
OC			
Yes	1 (7.1%)	13 (92.9%)	0.70
No	37 (14.5%)	219 (85.5%)	
Prior treatment			
Yes	2 (8%)	23 (92%)	0.74
No	36 (14.7%)	209 (85.3%)	
Surgeon volume			
High	24 (12.7%)	165 (87.3%)	0.054
Low	14 (17.3%)	67 (82.7%)	

Data are presented as number (percentage per column). The chi-square test was used for univariate analysis. Abbreviations: persist or rec, persistence or recurrence; prior treatment, prior treatment for CIN; OC, use of oral contraception.

**Table 3:** Comparison of patients’ characteristics and treatment outcome according to surgeon volume.

	High	Low	*p*-Value
	*n* = 189	*n* = 81	
Age	36 (18–60)	36 (21–53)	0.56
BMI	20.1 (14.8–31.1)	19.7 (16.2–26.7)	0.44
Parity			0.25
0	135 (71.4%)	52 (64.2%)	
≥1	54 (28.6%)	29 (35.8%)	
HPV			0.29
Positive	131 (69.3%)	27 (33.3%)	
Negative	4 (2.1%)	2 (2.5%)	
Not examined	54 (28.6%)	52 (64.2%)	
Smoking			0.23
Yes	89 (47.1%)	31 (38.3%)	
No	100 (52.9%)	50 (61.7%)	
OC			0.56
Yes	11 (5.8%)	3 (3.7%)	
No	178 (94.2%)	78 (96.3%)	
Prior treatment			0.64
Yes	19 (10.1%)	6 (7.4%)	
No	170 (89.9%)	75 (92.6%)	
Complications	11 (5.8%)	2 (2.5%)	0.36
Persist or rec	24 (12.7%)	14 (17.3%)	0.34

Data are presented as number (percentage per group) or median (range). The chi-square test was used for univariate analysis. Abbreviations: HPV, human papillomavirus; OC, use of oral contraception; prior treatment, prior treatment for CIN; persist, persistence; rec, recurrence.

**Table 4:** Univariate and multivariate analysis of predictive factors for persistence or recurrence.

		Univariate		Multivariate	
		HR (95% CI)	*p*-Value	HR (95% CI)	*p*-Value
Age	<50	1	0.83	1	0.64
	≥50	1.12 (0.40–3.17)		1.29 (0.45- 3.69)	
BMI	≤25	1	0.47	1	0.44
	>25	0.48 (0.066–3.53)		0.45 (0.062–3.69)	
Surgeon volume	Low	1	0.67	1	0.68
	High	0.86 (0.47–1.68)		0.87 (0.44–1.69)	
Prior treatment	No	1	0.24	1	0.21
	Yes	0.42 (0.10–1.76)		0.40 (0.096–1.69)	

Cox proportional hazards model. Abbreviations: prior treatment, prior treatment of CIN; HR, hazard ratio; CI, confidence interval; BMI, body mass index.

## Data Availability

All the studies used in the literature review are published. The data of case presentation are available from the corresponding author upon reasonable request.
